# Histological vis – a – vis biochemical assessment on the toxic level and antineoplastic efficacy of a synthetic drug Pt – ATP on experimental animal models

**DOI:** 10.1186/1756-9966-27-68

**Published:** 2008-11-12

**Authors:** Shipra Pal, Arpita Sengupta Sadhu, Swarup Patra, Kalyan K Mukherjea

**Affiliations:** 1 Department of Chemistry, Jadavpur University, Kolkata – 700032, India

## Abstract

**Background:**

Cisplatin, a platinum based anticancer drug has played a vital role in the treatment of cancers by chemical agents, but in view of the serious toxicity including nephrotoxicity of cisplatin, various other platinum based drugs have been synthesized and screened to overcome its toxicity. A Pt-ATP compound was prepared in our laboratory hoping to have reduced or no toxicity along with the potentiality of reducing neoplasm growth.

**Methods:**

A Pt-ATP compound was prepared. It was first screened for its antineoplastic efficacy. Confirming that, subsequent experiments were carried on to test its toxicity on animals, viz. Albino Swiss mice. The animals were randomly divided into four sets – Set I: Erhlich Ascites Carcinoma (EAC) challenged mice; Set II: Normal mice; Set III: Drug treated mice, Set IVA Cisplatin (CDDP) treated mice, Set IV B EAC challenged Cisplatin treated mice. Set I was used to test antineoplasticity of the drug, Set II and Set III for studying drug toxicity and Set IV was treated with CDDP. Set II was used as a control. Animals were sacrificed after 5 days, 10 days 15 days and 20 days of drug administration on the 6^th^, 11^th^, 16^th ^and 21^st ^days respectively for Set I, II and III. Set IVA was sacrificed only on the 16^th ^day and Set IV B on 6^th ^and 11^th ^days. For Set I only tumor cell count and packed cell volume (PCV) of tumor cells were recorded. For Set II and III, aspartate aminotransferase (AST), alanine aminotransferase (ALT) assays were done using serum while blood creatinine and creatine were assayed from blood filtrate. For cytotoxicity assessment liver, spleen and kidney tissues were collected and subjected to scanning electron microscopy (SEM) after extensive treatment. Set IV A was only studied for the biochemical parameters viz. aspartate aminotransferase (AST), alanine aminotransferase (ALT) assays were done using serum while blood creatinine and creatine were assayed from blood filtrate. Set IV B was studied for tumor cell count after treatment with CDDP for 10 days.

**Results:**

Our comparative studies with normal and drug treated animals reveal that the drug does not affect the body weight of the drug treated animals significantly. The biochemical parameters like ALT and AST levels are also within normal limits which rules out hepatotoxicity. The detailed histological studies by SEM reveal that the hepatic, kidney and spleen tissues are not adversely affected by the drug. Comparison of biochemical parameters with the CDDP treated animals show that Pt-ATP is not at all toxic like the CDDP. The Kaplan-Meier analysis of the survival data of Set I has shown promising results with a significance of p < 0.0001.

**Conclusion:**

Set I results are promising and indicating antineoplastic efficacy of the synthesized drug with increased life span of the animals. Biochemical analysis, hematological and SEM studies revealed that the drug was neither nephrotoxic nor hepato-spleeno-toxic under the experimental set up.

## Background

Neoplasm is any new and abnormal growth; specifically a new growth of tissue in which the growth is uncontrolled and progressive. The result is that they typically pile-up into a non structured mass or tumor. Neoplasm is of two types: malignant and benign. Malignant neoplasms are distinguished from benign in that the former shows a greater degree of anaplasia and have the properties of invasion and metastasis. This malignant neoplasm is termed cancer [1]. Cancer, one of the dreaded diseases of this present time is the cause of great concern to the modern society.

After the discovery of the anticancer activity of cisplatin Platinum based drugs have played a vital role in the treatment of cancers by chemical agents. Cisplatin cis-diaminedichloroplatinum (II) (DDP) has a wide variety of application in cancer chemotherapy [2-5]. Although cisplatin has been found to shrink the tumor burden it also results in toxicity by decreasing the body weight [6]. It is also extremely toxic to all fast proliferating normal cells [7,8]. The result of chemotherapy by cisplatin has also been reported to be unsatisfactory due to acquisition of chemoresistance by tumor cells [9]. Keeping in view the serious toxicity including nephrotoxicity of these drugs, various other platinum based drugs have been synthesized and screened to overcome toxicity of cisplatin [10-13].

ATP and its metal complexes control bioenergetics of physiological systems. So, we have tried to develop a novel platinum complex with the natural constituent ATP hopefully with improved potency and less toxicity to the hosts. The synthetic complex Pt – ATP has been shown to demonstrate anticancer activity in experimental animal model [14]. When treated with the drug, it has been found to decrease the tumor cell count in the animals challenged with Erhlich Ascites Carcinoma cells compared to the untreated control group of animals. As this drug warrants a detailed study on the level of toxicity, our present investigation emphasizes the study of toxicity of the drug on normal animals. The results of our experiments are being presented in the present communication.

## Methods

### Animals

Male Swiss albino mice were used for the experiment. The mice were kept in ventilated cages and maintained on normal diet and water. The mice were divided into three sets and challenged with appropriate neoplastic cell lines to develop neoplasm.

1. **Set I **– An aliquot of 0.5 ml containing 0.5 × 10^6 ^Erhlich Ascites Carcinoma (EAC) cells were transplanted into the peritoneal cavity of Albino Swiss mice and experiments were carried out. These mice were again divided into three groups. Set I A – tumor control; Set I B – low dose drug treated and Set I C – high dose drug treated.

2. **Set II **– Normal mice.

3. **Set III **– Drug treated mice further divided into two groups: Set III A – Low dose drug treated group and Set III B – High dose drug treated group.

4. **Set IV **– Cisplatin cis-dichlorodiammineplatinum (II) complex treated animals.

Set IV A, only CDDP treated group, Set IV B EAC challenged mice treated with CDDP.

### Preparation of the Pt-ATP compound

The compound (drug) was synthesized following our earlier methods described previously [15]. K_2_PtCl_4 _was purchased from CDH, India and 5'-adenosine triphosphate disodium salt was purchased from SRL, India. All other chemicals were AR or GR grade. All glass triple distilled water was used throughout.

### Treatment schedule and experimental design

The compound was dissolved in normal saline (0.9% NaCl in distilled water, pH = 7.4) immediately before use and was injected intraperitonially with a dose of 10 mg per kg body weight per day for low dose drug treated group (Set I B & Set III A) and 20 mg. per kg body weight per day for high dose drug treated group (Set I C & Set III B) for 10 consecutive days. Set IV A animals were treated with CDDP with a dosage of 10 mg/kg body weight for 10 consecutive days and animals sacrificed on the 16^th ^day. Set IV B animals were challenged with EAC (0.5 × 10^6 ^cells), treated with 10 mg/Kg body weight of CDDP and sacrificed on 6^th ^and 11^th ^days. Animals were sacrificed by cardiac puncture under general anesthesia (GA) using thiopental after 5 days, 10 days 15 days and 20 days of drug administration i.e. on the 6^th^, 11^th^, 16^th ^and 21^st ^days respectively. Tumor cell count and packed cell volume (PCV) were noted for Set I. For Set II and Set, III blood was collected by cardiac puncture and serum extracted from it by centrifugation. Tissues were stored in 2.5% gluteraldehyde at 4°C for scanning electron microscope (SEM) studies. For Set IVA blood was collected, serum collected and blood filtrate prepared as described above after sacrifice of animals. For Set IV B only tumor cell count was noted.

### Tumor cell count

Tumor cell counts were done with the help of hemocytometer by the use of trypan blue exclusion method.

### PCV estimation

The ascetic fluid was collected after sacrifice from the peritoneal cavity with the help of a Pasteur pipette. The peritonial cavity was washed with PBS and the tumor cells suspended in the PBS. It was then centrifuged at 1500 rpm for 5 minutes at room temperature in a graduated centrifuge tube. The Cell volume thus precipitated after centrifugation was recorded.

### Anthropometric studies

Body weights of the mice were recorded initially and at regular intervals of 5 days i.e. on the days of sacrifice. Weights of the tissues viz. liver, kidney and spleen were also noted and were expressed as average percentage of total body weight.

### Toxicity studies

#### Biochemical toxicity assessment

Estimation of aspartate aminotransferase (AST), alanine aminotransferase (ALT) activity, blood creatinine and blood creatine were done by following spectrophotometric method [16].

#### Cytotoxicity assessment

Kidney, liver and spleen were cut into small cubes of 2 mm dimension and fixed in Osmium tetroxide solution. Those fixed tissues were subjected to CPD (critical point drying) after extensive dehydration process, then gold coated and observed under SEM.

### Suvivality studies

Kaplan-Meier curve was drawn for survivality analysis with the help of MedCalc software [17,18].

Statistical analyses were done using Microsoft Excel while images were adapted by Adobe Photoshop.

## Results

### Set I

**Figure 1:**
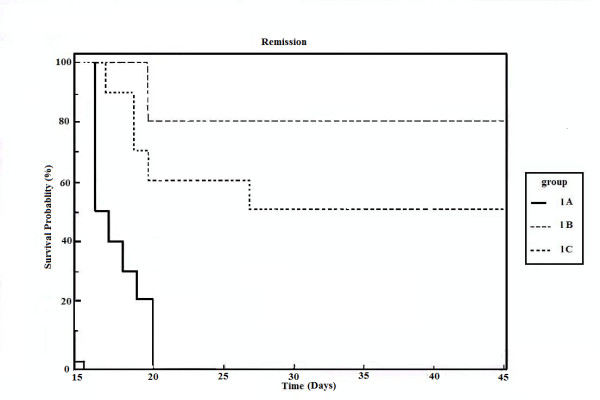
Kaplan-Meier curve of survival data of Set I animals.

The tumor cell counts of the animals bearing EAC cells with and without drug treatment are noted in Table 1. Table 2 represents the PCV of Set I animals. Figure 1 represents the Kaplan-Meir curve for survivality analysis of the Set I animals.

**Table 1:** Total tumor cell count of EAC bearing animals.

Time	TUMOR CONTROL (× 10^6^)	Dose I* (× 10^6^)	Dose II ** (× 10^6^)
Passage	0.5 ± 0.00	0.5 ± 0.00	0.5 ± 0.00

After 5 days	1.0 ± 0.39	0.7 ± 0.16	0.7 ± 0.16

After 10 days	2.2 ± 0.14	1.7 ± 0.07	1.3 ± 0.05

* Dose I = 10 mg per Kg body weight

** Dose II = 20 mg per Kg body weight

Data represented as mean value ± S.D

**Table 2:** PCV values in mL^# ^of EAC bearing animals.

Time	TUMOR CONTROL	Dose I	Dose II
After 5 days	0.01 ± 0	Immeasurable quantity	Immeasurable quantity

After 10 days	0.125 ± 0.050	0.0625 ± 0.025	0.125 ± 0.050

N = 3. (mean ± S.D)

^# ^Measured in μL

Data represented as mean value ± S.D

### Set II & III

The results of change in body weight of the animals are presented in Table 3, while the weights of the kidney, liver and spleen of the animals are presented in Tables 4, 5, and 6 respectively. The weights of the kidney, liver and spleen are presented as percentage weight of the animals' body weight for the sake of better comparison.

**Table 3:** The average body weight of animals in grams.

**TIME**	**NORMAL**	**LOW DOSE DRUG TREATED**	**HIGH DOSE DRUG TREATED**
Initial	14.9 ± 1.5	19.1 ± 1.6	21.9 ± 2.7

After 5 days	18.4 ± 1.6	21.6 ± 1.9	24.2 ± 5.0

After 10 days	18.3 ± 1.8	20.8 ± 2.5	22.3 ± 4.0

After 15 days	18.5 ± 1.6	19.6 ± 1.9	22 ± 3.1

After 20 days	19.5 ± 1.2	20.6 ± 2.4	23.2 ± 3.8

Data represented as mean value ± S.D

**Table 4:** The weight of kidney as percentage of total body weight.

**TIME**	**NORMAL**	**LOW DOSE DRUG TREATED**	**HIGH DOSE DRUG TREATED**
After 5 days	1.3 ± 0.4	1.0 ± 0	1.1 ± 0.1

After 10 days	1.0 ± 0	1.0 ± 0	1.4 ± 0.3

After 15 days	1.0 ± 0	1.3 ± 0.1	1.3 ± 0.1

After 20 days	1.0 ± 0	1.1 ± 0.2	1.1 ± 0.1

Data represented as mean value ± S.D.; n = 3

**Table 5:** The weight of liver as percentage of total body weight of the animals.

**TIME**	**NORMAL**	**LOW DOSE DRUG TREATED**	**HIGH DOSE DRUG TREATED**
After 5 days	4.5 ± 0.7	4.8 ± 0.9	4.9 ± 0.9

After 10 days	5.0 ± 0	5.2 ± 0.7	6.3 ± 0.6

After 15 days	4.5 ± 0.7	5.3 ± 0.5	5.3 ± 0.6

After 20 days	5.9 ± 0.2	4.7 ± 0.8	5.1 ± 0.8

Data represented as mean value ± S.D.; n = 3

**Table 6:** The weight of spleen as percentage of total body weight of the animals.

**TIME**	**NORMAL**	**LOW DOSE DRUG TREATED**	**HIGH DOSE DRUG TREATED**
After 5 days	0.4 ± 0	0.5 ± 0.2	0.3 ± 0.1

After 10 days	0.4 ± 0.1	0.4 ± 0.1	0.4 ± 0.1

After 15 days	0.5 ± 0.1	0.3 ± 0.1	0.5 ± 0.1

After 20 days	0.4 ± 0.1	0.3 ± 0.1	0.4 ± 0.2

Data represented as mean value ± S.D.; n = 3

To ascertain the level of toxicity of the platinum drug, a detailed biochemical investigation was done. The results of the biochemical investigation are presented in Table 7 (blood creatinine), Table 8 (blood creatine), and Table 9 (ALT and AST).

**Table 7:** The average values of blood creatinine in mg/dL of different group of animals.

**TIME**	**NORMAL**	**LOW DOSE DRUG TREATED**	**HIGH DOSE DRUG TREATED**
After 10 days	0.53	0.29	0.29

After 15 days	0.31	0.33	0.55

After 20 days	0.28	0.18	0.31

**Table 8:** The average values of blood creatine in mg/dL of different group of animals.

**TIME**	**NORMAL**	**LOW DOSE DRUG TREATED**	**HIGH DOSE DRUG TREATED**
After 10 days	1.07	0.76	0.88

After 15 days	0.52	0.52	0.64

After 20 days	1.08	1.08	1.78

**Table 9:** The average values of ALT/AST in I.U of different group of animals.

**TIME**	**NORMAL**		**LOW DOSE DRUG TREATED**		**HIGH DOSE DRUG TREATED**	
	ALT	AST	ALT	AST	ALT	AST

After 15 days	88.9	71.67	72.2	55.9	67.3	67.6

After 20 days	66.9	100.1	64.2	83.5	64.26	34.7

Kidney, Liver and spleen tissues were subjected to extensive histological studies under Scanning Electron Microscope. The results with each type of tissue in Normal (without any drug), Low dose and High dose of drugs are presented with respective captions Figures (2, 3, 4, 5, 6, 7, 8, 9, 10).

**Figure 2:**
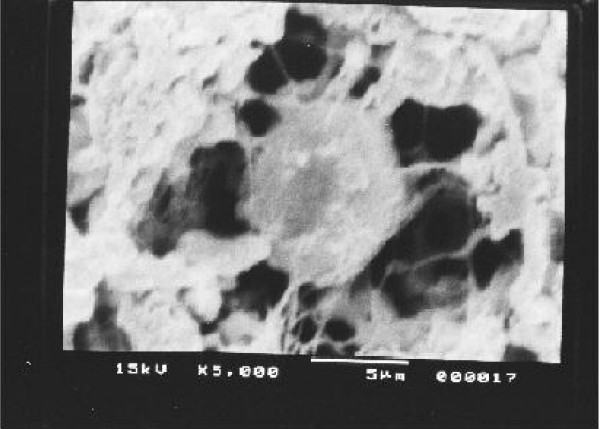
Scanning electron micrograph of normal hepatocyte.

**Figure 3:**
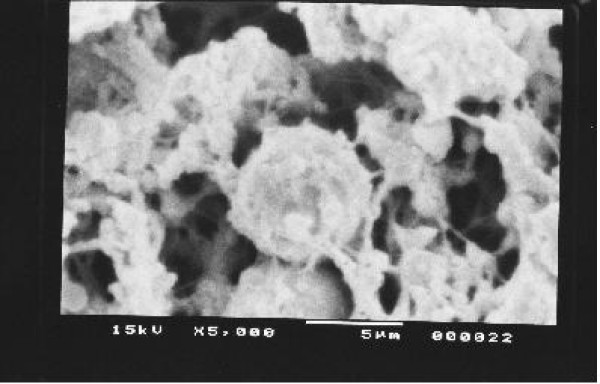
Scanning electron micrograph of drug treated hepatocyte (low dose).

**Figure 4:**
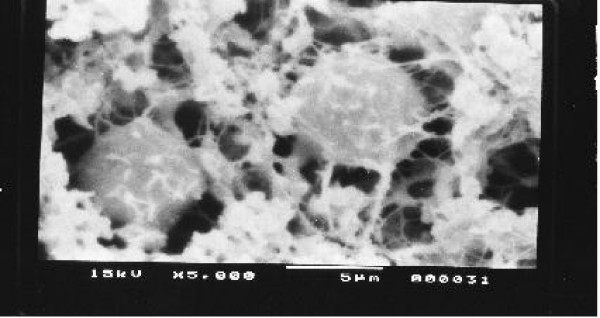
Scanning electron micrograph of drug treated hepatocyte (high dose).

**Figure 5:**
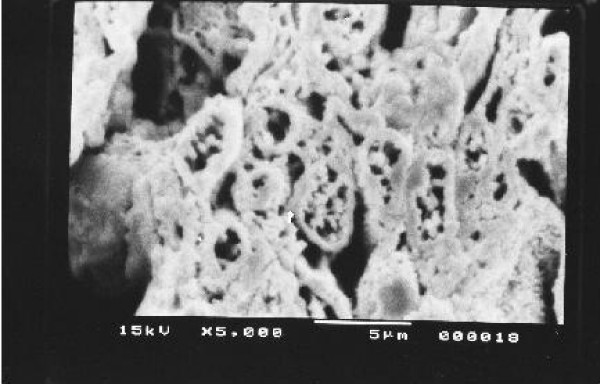
Scanning electron micrograph of normal kidney medulla.

**Figure 6:**
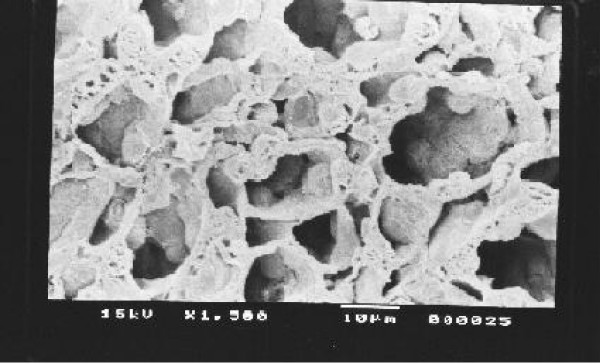
Scanning electron micrograph of drug treated kidney medulla (low dose).

**Figure 7:**
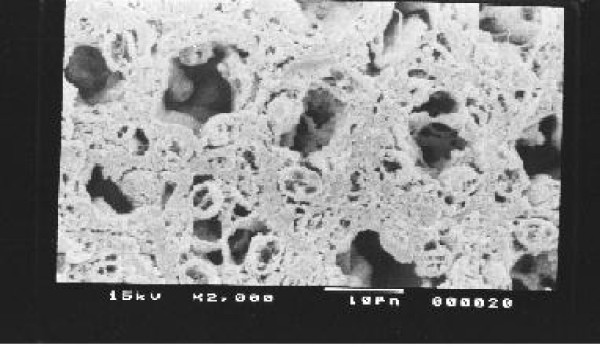
Scanning electron micrograph of drug treated kidney medulla (high dose).

**Figure 8:**
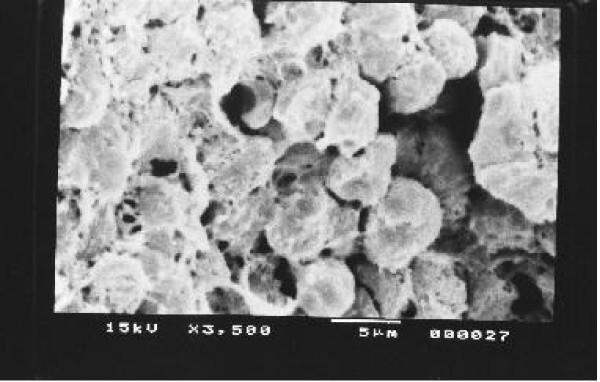
Scanning electron micrograph of normal spleen.

**Figure 9:**
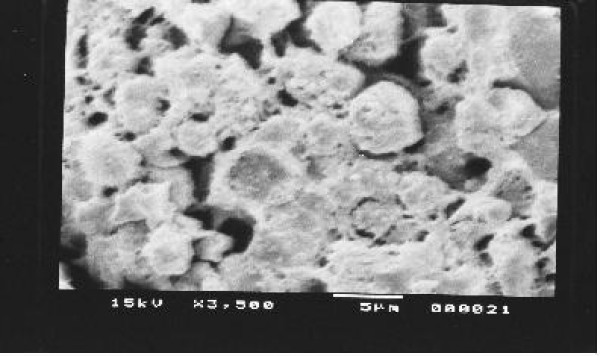
Scanning electron micrograph of drug treated spleen (low dose).

**Figure 10:**
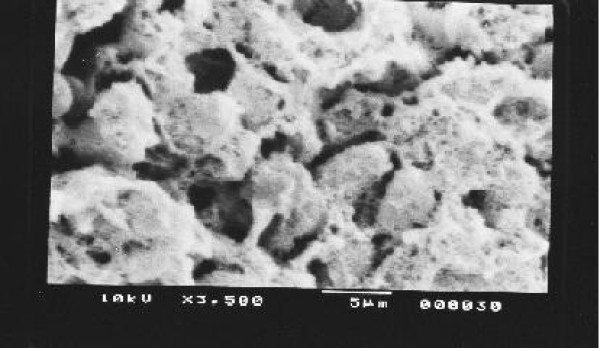
Scanning electron micrograph of drug treated spleen (high dose).

### Set IV

The biochemical parameters of CDDP treated animals were noted (Table 10) for the comparison of our Pt-ATP cisplatin analogue with CDDP. Table 11 represents the tumor cell count of EAC challenged CDDP treated animals.

**Table 10:** Biochemical parameters of CDDP treated animals.

**TIME**	ALT (I.U)	AST (I.U)	Blood Creatinine (mg/dL)	Blood Creatine (mg/dL)
After 15 days	110.9	184.5	13.01	16.05

**Table 11:** Tumor cell count of CDDP treated animals.

Time	TUMOR CONTROL (× 10^6^)	CDDP treated mice (× 10^6^)
Passage	0.5 ± 0.00	0.5 ± 0.00

After 5 days	1.0 ± 0.39	0.6 ± 0.16

After 10 days	2.2 ± 0.14	1.6 ± 0.15

## Discussions

Our previous preliminary studies indicated that the synthetic platinum compound Pt-ATP exhibited antineoplastic efficacy in EAC and Sarcoma 180 cells [14]. Set I results (Table 1 and 2) in the present study also reflect the results of our previous studies. The survivality analysis with the help of Kaplan-Meier curve (Fig 1) shows an increase in the survivality of the animals treated with Pt-ATP which is seen to increase by as much as 50% in case of Set IC whereas it is as high as 80% in case of Set IB animals. The significance of level is p < 0.0001 according to the Chi Square test. The control group (Set I) is seen to die within 20 days whereas the treated groups remain alive for 45 days and more. As a number of platinum drugs are known to impart high level of toxicity on the drug treated hosts, the development of anticancer platinum drugs with lower degree of toxicity continued. We also synthesized the said Pt-ATP drug with a naturally occurring ligand ATP considering that this may be less toxic to the hosts. So, in this work we have undertaken a detailed investigation on the toxic levels. Our comparative studies with normal and drug treated animals reveal that the drug does not affect the body weight of the drug treated animals significantly. Many anticancer drugs are known to affect the kidney, liver and spleen adversely, so we have undertaken studies by monitoring the relative weights of these drug treated organs with normal ones and found that they are very much within normal limits in the drug treated groups. The biochemical parameters like ALT and AST levels are also within normal limits which rule out hepatotoxicity. The normal levels of blood creatinine and blood creatine indicate that the drug does not impart any adverse effect on the kidney of the hosts. The detailed histological studies by SEM reveal that the hepatic cells are not adversely affected by the drug (Figs 3, 4). The kidney medullas are visualized with distinct clarity (Figs 6, 7) which indicates that the drug is not nephrotoxic either. The spleenic cellularity has clearly been demonstrated in the drug treated animals both at higher and lower dose of the drug (Figs 9, 10). Set IV A results (Table 10) clearly depict an increase in serum AST and ALT levels indicating hepatotoxicity to a mild extent. It also shows a dramatic increase in blood creatine and creatinine values indicating nephrotoxicity. Thus, even though the Set IV B results represent a pleasant picture with the antitumor activity but due to its serious toxic effects, its use as an anticancer drug needs reviewing. In comparison to our results of Set II and Set III, CDDP appears to be toxic to the animals.

Our above studies reveal that this drug does not induce any apparent toxicity on the treated hosts and seems to be a promising drug in the management of cancer under the present experimental set up.

Further studies both with respect to toxicity and dose response activity in cancerous animals are underway in our laboratory.

## Conclusion

The results of Set I clearly establishes the antineoplastic efficacy of the synthesized Pt – ATP drug. The biochemical analysis of the ALT, AST, blood creatinine and blood creatine levels reveal that the above values are within the normal range. SEM observation shows normal cell morphology of the tissues of all groups. Results of Set IV also show that although CDDP is an effective anticancer drug it has serious toxic effects on the hosts. This study suggests that the present drug, the Pt-ATP neither being hepato-spleeno-toxic nor nephrotoxic at the present experimental set up has a bright prospect for future studies.

## Authors' contributions

S Pal and AS contributed equally to the work. S Pal, AS and S Patra executed the experimental work; while KKM planned and designed the experiment, guided the work, took part in critical analysis of the results and prepared the present manuscript.
